# Risk factors for and treatment of anastomotic strictures after Ivor Lewis esophagectomy

**DOI:** 10.1007/s00464-024-11150-w

**Published:** 2024-08-19

**Authors:** Sophie L. F. Doran, Maria G. Digby, Sophie V. Green, Clive J. Kelty, Anand P. Tamhankar

**Affiliations:** 1 Department of Upper Gastrointestinal Surgery, Sheffield Teaching Hospitals NHS Foundation Trust, Sheffield, S5 7AU UK; 2 Academic Unit of Surgery, University of Sheffield, Northern General Hospital, Herries Road, Sheffield, S5 7AU UK

**Keywords:** Esophagectomy, Anastomotic stricture, Weight loss

## Abstract

**Introduction:**

Anastomotic strictures following esophagectomy occur frequently and impact on nutrition and quality of life. Although strictures are often attributed to ischemia and anastomotic leaks, the role of anastomosis size and pyloroplasty is not well evaluated. Our study aims to assess the rate of and risk factors for anastomotic stricture following esophagectomy, and the impact of treatment with regular endoscopic balloon dilatations.

**Methods:**

Consecutive patients (*n* = 207) undergoing Ivor Lewis esophagectomy performed by two surgeons at our institution were included. Data on patient demographics, surgical outcomes and anastomotic strictures were recorded. Relationship of anastomotic strictures with circular stapler size, pyloroplasty and anastomotic leak was analyzed. Treatment of strictures with endoscopic balloon dilatation was reviewed and percentage weight loss at 1 year was evaluated.

**Results:**

Anastomotic strictures occurred in 17.4% of patients. Patient demographics between those with and without stricture were similar. Stricture rate was similar in patients with or without pyloroplasty (13.9% vs 21.7%, respectively, *p* = 0.14) and in those with or without an anastomotic leak (25.0% vs 16.6%, respectively, *p* = 0.345). Stricture risk increased with smaller sized stapler (25 mm = 33.3%, 28 mm = 15.3%, 31 mm = 4.8%; *p* = 0.027).

The median number of dilatations required to fully treat strictures was 2 (IQR: 1–3). The median length of time from surgery to first dilatation was 2.9 months (IQR: 2.0–4.7) and to last dilatation was 6.1 months (IQR: 4.8–10.0). Median maximum dilatation diameter was 20 mm (IQR: 18.0–20.0). There were no complications from dilatations. Percentage weight loss at 1 year in patients with strictures was similar to those without strictures (8.7% vs 11.1%, respectively, *p* = 0.090).

**Conclusions:**

Post-esophagectomy anastomotic strictures are common and not necessarily related to anastomotic leaks or absence of pyloroplasty. Smaller anastomosis size was strongly linked with stricture formation. A driven approach with regular endoscopic balloon dilation is safe and effective in treating these strictures with no excess weight loss at 1 year once treated.

Ivor Lewis esophagectomy is the most commonly employed surgical treatment for potentially curable esophageal carcinoma. Despite improvements in operative technique and standardized post-operative protocols there remains a high morbidity rate of up to 60% related to an Ivor Lewis esophagectomy with subsequent impact on quality of life [1, 2]. One such troublesome complication of an Ivor Lewis esophagectomy is an anastomotic stricture with a reported incidence of 18–42% [3–5].

An anastomotic stricture following an Ivor Lewis esophagectomy refers to a narrowing at the site of the esophago-gastric anastomosis. The cardinal symptom of an anastomotic stricture following an esophagectomy is dysphagia. Other presenting symptoms may include food bolus obstruction, aspiration pneumonia or excess weight loss following esophagectomy.

Anastomotic strictures following esophagectomy are diagnosed by contrast radiological studies or by endoscopy. The aim of endoscopic intervention is to achieve an esophageal lumen ≥ 15mm although this is a largely arbitrary cut-off without an evidence base [6]. The optimum size will vary between patients and should be individualized.

Different techniques exist to create esophago-gastric anastomosis during an Ivor Lewis esophagectomy including circular stapled, linear stapled and a hand-sewn anastomotic technique. However across multiple studies one anastomotic technique has not been shown to be superior over another [7–11]. Several studies have looked into risk factors for the development of anastomotic strictures including conduit ischemia, anastomotic leaks, co-morbidities and neoadjuvant treatment. Few studies have considered the impact of pre-operative weight, anastomosis size and the use of pyloric drainage procedures such as pyloroplasty during an esophagectomy on anastomotic stricture rate. Also little is known about the long-term nutritional impact of anastomotic strictures and their treatment.

The aim of this study was to evaluate the rate of and risk factors for anastomotic stricture following esophagectomy using a standardized circular stapled technique and to assess the impact of treatment with endoscopic balloon dilatation on post-operative weight loss.

## Methods

### Study design

This was a retrospective analysis of a contemporaneously maintained database at a regional upper gastro-intestinal cancer center in the United Kingdom. All patients who underwent an Ivor Lewis esophagectomy for adenocarcinoma or squamous cell carcinoma of the esophagus or gastro-esophageal junction between April 2013 and April 2021 were included. Data collection included patient demographics (age, gender, ASA (American Society of Anesthesiologists) grade, neoadjuvant treatment and pre-operative weight). Post-operative histological parameters were collected including tumor subtype (adenocarcinoma or squamous cell carcinoma), tumor, lymph node, metastasis, and resection margin stage according to the 8th edition of the American Joint Committee on Cancer (AJCC) staging of epithelial cancers of the esophagus and esophagogastric junction. For subsequent analysis patients were divided into two groups including: those that did not develop an anastomotic stricture and those that did have an anastomotic stricture. Anastomotic strictures were identified in the presence of dysphagia and stenosis at the anastomosis on oral contrast studies or endoscopy and required endoscopic dilatation.

Clinical outcomes including 30- and 90-day mortality rate, anastomotic leak rate, respiratory complication rate and percentage weight loss at 1 year were collected. The presence of pyloroplasty and the size of anastomosis was recorded.

Patients identified with an anastomotic stricture were treated with endoscopic anastomotic balloon dilatation. Data were collected on the number of endoscopic anastomotic balloon dilatations required, the diameter of the balloon used for dilatation and the time interval from surgery to endoscopic intervention.

### Surgical technique

Each patient underwent clinical staging using a combination of endoscopy, computed tomogram and positron emission tomogram as is the standard at our institution. Neoadjuvant therapy with chemotherapy or chemoradiotherapy was given to all patients with node-positive disease and/or ≥ T2 disease. Resection was performed 5–8 weeks following completion of neoadjuvant therapy. All operations were performed by one of the two surgeons (denoted surgeon A and surgeon B) within the Department of Upper Gastrointestinal Surgery.

A standard Ivor Lewis esophagectomy was performed using trans-abdominal and right thoracotomy access. A complete lymphadenectomy of the celiac branches was performed. The duodenum was Kocherized. The stomach and distal esophagus were mobilized to above the hiatus. At the end of the abdominal phase surgeon A did not routinely perform a pyloroplasty and surgeon B routinely did perform a pyloroplasty. During pyloroplasty the entire muscle and gastric mucosa at the pylorus were divided longitudinally. The pylorotomy was then closed transversely in a single layer with interrupted 3–0 polydioxanone sutures to create a Heineke-Mikulicz type pyloroplasty. Feeding jejunostomies were not routinely inserted. Thoracic esophageal mobilization and lymphadenectomy was then performed via a right posterolateral approach. Azygos arch was divided and thoracic duct ligated above the diaphragm. The stomach was tubularized to create a conduit approximately 5 cm in width and a subsequent intrathoracic anastomosis using a circular stapler was performed above the level of azygos arch. A standard circular stapled end-to-side esophago-gastric anastomosis was performed using a Medtronic EEA™ DST™ stapler with diameters 25 mm, 28 mm or 31 mm. The size of the stapler used depended on the available diameter of the proximal esophagus. The largest size of circular stapler was used based on the available diameter of the proximal esophagus. The largest size of stapler head (25 mm, 28 mm or 31 mm) was used which could be inserted without tearing the proximal esophagus. The proximal esophagus was routinely dilated prior to stapler head insertion with a 28 Fr Foley catheter balloon, to gently stretch the lumen and reduce spasm to allow the largest stapler head to fit in. The circular staple line was not oversewn but was wrapped with surrounding available omentum.

All patients were managed according to a standardized post-operative protocol. All patients received proton pump inhibitor therapy post-operatively and this was continued on a long-term basis orally after discharge.

### Definitions

#### Mortality rate

Patients who did not survive 30-day or 90-day time points were identified for mortality rate assessment.

#### Anastomotic leak

Anastomotic leak was diagnosed by oral contrast study and computed tomogram following clinical suspicion as indicated by fever and / or leucocytosis or rising C reactive protein level.

#### Respiratory complications

Respiratory complications, including pneumonia (diagnosed by a combination of clinical symptoms suggestive of the diagnosis, leukocytes and infiltrates on imaging), were recorded if classified as Clavien-Dindo grade ≥ 3.

#### Anastomotic stricture

Anastomotic stricture was identified in the presence of dysphagia and stenosis at the anastomosis on oral contrast studies or endoscopy and required endoscopic dilatation.

#### Post-operative anastomotic stricture endoscopic treatment

Patients who had post-operative dysphagia were offered endoscopic assessment or contrast swallow study. Those found to have stenosis at the anastomosis were offered endoscopic balloon dilatation. Endoscopic balloon dilatation was performed under sedation with a through-the-endoscope balloon (Boston Scientific CRE™ balloon dilator), with the size employed dependent on the stricture and the endoscopist’s judgement. Patients whose maximum dilation size was ≤ 12 mm were offered repeat endoscopic dilatations in two weeks. Those with maximum dilatation size of ≤ 15mm were offered repeat dilatations in four weeks. Dilatations were stopped once dysphagia was resolved, and maximum dilatation size was ≥ 15mm.

#### Percentage weight loss

Weight loss at a 1-year time point was compared to immediate pre-operative weight (following any neoadjuvant treatment) to calculate percentage weight loss.

### Exclusion criteria

Patients were excluded from analysis if they underwent an Ivor Lewis esophagectomy for a diagnosis other than adenocarcinoma or squamous cell carcinoma of the esophagus or gastro-esophageal junction. For the evaluation of percentage weight loss at 1-year post-operatively, patients were excluded if they had been diagnosed with recurrence or died prior to the 1-year timepoint. Anastomotic strictures which occurred due to disease recurrence were excluded from the study.

### Statistical analysis

Demographic data were summarized and compared between the groups using the Mann–Whitney *U-*test for continuous variables and Chi-Squared test for categorical variables. For all tests a two-sided p value of 0.05 was deemed to be significant. All statistical analysis was conducted on DATAtab: Online Statistics Calculator (DATAtab e.U. Graz, Austria).

### Ethics and consent

Data analyzed in this study were collected from a contemporaneously maintained database from the Upper Gastrointestinal Surgical unit at our institute. Data review was approved by Local Clinical Effectiveness Unit (CEU project registration number: 11763). No patient identifying information was recorded and patient consent was not required for data review as per CEU guidelines.

## Results

During the study period 207 patients met the inclusion criteria. 171 patients (82.6%) did not have an anastomotic stricture and 36 patients (17.4%) had an anastomotic stricture. 57 patients were excluded from the analysis of weight loss at 1 year, 5 of these patients had disease recurrence at the anastomosis. There was no statistically significant difference between the groups in terms of age, gender, ASA grade, pre-operative weight, neoadjuvant treatment, tumor subtype, TNM stage or resection margin status. Of note all patients were classified as ASA grade 2 or 3 and all patients were M0 according to the TNM system. There were no R1 longitudinal or R2 resections. All results summarized in Table 1.

**Table 1:** Patient characteristics

	No anastomotic stricture (n = 171)	Anastomotic stricture (n = 36)	*p* value^*a*^
Age	67 (61–72)	65 (59–72)	0.458^b^
Gender			0.689
Male	138	28	
Female	33	8	
ASA Grade			0.0.248
2	127	30	
3	44	6	
Pre-operative weight (kg)	79.0 (70.1–90.1)	75.1 (67.0–88.0)	0.188^b^
Neoadjuvant treatment			0.747
Yes	114	25	
No	57	11	
Tumor Subtype			0.847
Squamous Cell Carcinoma	16	3	
Adenocarcinoma	155	33	
T stage			0.797
0–1	56	9	
2	23	6	
3	89	20	
4	3	1	
N stage			0.973
0	80	18	
1	45	9	
2	28	6	
3	18	3	
Resection margin			0.939
R0	132	28	
R1 (circumferential)	39	8	

Values are median (IQR), otherwise n

^a^Chi-Squared test except ^b^Mann–Whitney U–Test

### Analysis of etiology of anastomotic strictures and clinical outcomes

The anastomotic stricture rate in patients who had a pyloroplasty was similar to those without (13.9% vs 21.7%, respectively, *p* = 0.14). Anastomotic stricture rate was also similar in those patients who had an anastomotic leak to those who did not (25.0% vs 16.6%, respectively, *p* = 0.345).

A standard circular stapled end-to-side esophago-gastric anastomosis was performed using a Medtronic EEA™ DST™ stapler with diameters 25mm, 28mm or 31mm. 33.3% of patients with anastomosis size 25mm developed an anastomotic stricture, 15.3% of patients with anastomosis size 28mm developed an anastomotic stricture and 4.8% of patients with anastomosis size 31mm developed an anastomotic stricture. There was a statistically significant trend between decreasing anastomosis size and the development of an anastomotic stricture (*p* < 0.05). Further analysis revealed that there was a statistically significant risk of anastomotic stricture when the anastomosis size was 25 mm versus greater than 25mm (28 or 31 mm) (*p* < 0.05). However such risk was not evident between anastomosis sizes 25 or 28 mm versus 31 mm (*p* > 0.05) suggesting that anastomosis size 25mm is most significantly linked with anastomotic stricture formation. These results are detailed in Table 2.

**Table 2:** Anastomosis size

	No anastomotic stricture (*n* = 171)	Anastomotic stricture (*n* = 36)	*p* value^*a*^
Anastomosis size			0.027*
25 mm (*n* = 36)	24 (66.7%)	12 (33.3%)	
28 mm (*n* = 150)	127 (84.7%)	23 (15.3%)	
31 mm (*n* = 21)	20 (95.2%)	1 (4.8%)	

^a^Chi-Squared test. *Denotes statistical significance at 0.05 level

Other clinical outcomes including 30- and 90-day mortality rate were similar in both groups. Respiratory complications (Clavien-Dindo grade ≥ 3) were higher in the no anastomotic stricture group. Percentage weight loss at 1-year post-operatively was also similar in both groups. These results are summarized in Table 3.

**Table 3:** Clinical outcomes

	No anastomotic stricture (*n* = 171)	Anastomotic stricture (*n* = 36)	*p value*^a^
30-day mortality	4 (2.3%)	0 (0.0%)	0.354
90-day mortality	8 (4.7%)	0 (0.0%)	0.186
Respiratory complications (CD ≥ 3)	19 (11.1%)	0 (0.0%)	0.036*
Weight loss at 1 year	11.1% (5.8–17.3, *n* = 118)	8.7% (3.3–13.1, *n* = 32)	0.090^b^

Values are median (IQR), otherwise n

^a^Chi-Squared test except, ^b^Mann–Whitney U–Test. *Denotes statistical significance at 0.05 level

### Endoscopic balloon dilatation of anastomotic strictures

A total of 36 patients (17.4%) developed an anastomotic stricture requiring endoscopic balloon dilatation. Of those patients, 12 (33.3%) required only one endoscopic balloon dilatation and 24 patients (66.7%) required more than one. The median number of dilatations required to fully treat anastomotic strictures was 2 (IQR 1–3). The median length of time from surgery to first dilatation was 2.9 months (IQR 2.0–4.7) and to last dilatation was 6.1 months (IQR 4.8–10.0). Median maximum dilatation balloon diameter of the first dilatation was 18.0mm (IQR 16.5–20.0). Patients were offered a further endoscopic balloon dilatation in 2 weeks if the maximum endoscopic balloon diameter reached was ≤ 12 mm, and a further dilatation within 4 weeks if the maximum endoscopic balloon diameter reached was ≤ 15 mm. Median maximum balloon diameter of the last dilatation was 20.0 mm (IQR 18.0–20.0). Repeated endoscopic balloon dilatations were performed until resolution of dysphagia. There were no complications including bleeding, perforation and aspiration resulting from endoscopic anastomotic balloon dilatation.

## Discussion

There is a large variation in the incidence of anastomotic strictures (18–42%) following Ivor Lewis esophagectomy [3, 5, 12, 13]. We believe this variation is due to heterogenicity in the definition of an anastomotic stricture. In our study anastomotic strictures were defined when they caused significant symptoms and required dilatation. We acknowledge that esophageal size varies in normal individuals. Furthermore, in some patients the tumors can be obstructing and may cause upstream esophageal widening. We do not believe in exposing all patients to radiation from contrast swallow studies to assess for true anastomotic size. Our approach was to identify clinically relevant patients with dysphagia and then subject them to further invasive testing and offer treatment if anastomotic size was narrow. With this approach our cohort had an overall stricture rate of 17.4%, and with effective endoscopic treatment no patients had excess weight loss at 1 year following surgery.

There are multiple factors that may contribute to the development of an anastomotic stricture. It is suggested that ischemia and tension at the anastomosis may contribute to the development of an anastomotic stricture [3]. Ischemia at the anastomotic site in the gastric conduit has been shown to be associated with an increased risk of anastomotic stricture formation [14] and gastric ischemic pre-conditioning has been shown to reduce anastomotic stricture formation [15]. Ischemia and tension at the anastomosis have also been linked to anastomotic leak which is a known risk factor for anastomotic stricture formation [3, 12, 13, 16]. Additionally, reflux of gastric contents is thought to lead to inflammation, collagen and fibrin deposition and subsequent scarring at the anastomotic site [17, 18]. Other factors such as patient age, gender, co-morbidities and the use of neoadjuvant radiotherapy have been suggested as being related to anastomotic stricture formation but the mechanisms that underly this are not well understood [12, 13, 19, 20].

Our study is a large single center study with patients operated on by only two surgeons which lends to uniformity in technique and reduction in variability in conduit vascularity and tension at the anastomosis. Our overall anastomotic leak rate was 9.7% which is comparable to most other series [8]. All patients received post-operative long-term proton pump inhibitors to reduce acid reflux. With this our anastomotic stricture incidence remains at the lower end of the reported literature.

Anastomotic leaks are often considered the index event in the development of an anastomotic stricture. Our study has shown that anastomotic stricture formation was higher in patients who developed an anastomotic leak (25% versus 16.6%), however this was not statistically significant. Certainly other factors must play a role in the development of anastomotic strictures.

In our cohort there was no relationship between age, gender, ASA grade or pre-operative weight and the development of an anastomotic stricture. Additionally there was no relationship between tumor-related factors (TNM stage, resection margin status, tumor subtype: squamous cell carcinoma or adenocarcinoma, or the use of neoadjuvant treatment and the development of an anastomotic stricture. We also assessed the role of pyloroplasty or its absence in the development of anastomotic strictures. There are no studies that assess the relationship of pyloric intervention to anastomotic stricture formation. It is more important to consider this as more esophagectomies are being performed as minimally invasive procedures and pyloroplasty is often not performed in such an approach. Proponents of pyloroplasty advocate that the pyloric denervation that is a result of the high thoracic truncal vagotomy leads to gastric dysmotility and delayed gastric emptying. This in turn can lead to a distended gastric conduit with tension on the anastomosis and anastomotic leakage. Additionally delayed gastric emptying could contribute to long-term reflux which can cause strictures. In our cohort we did not find that the presence or absence of a pyloroplasty had an impact on anastomotic stricture rate. As an esophagectomy is a complex, multi-step, multi-variable operation it is logical that the presence or absence of pyloroplasty alone with its variable impact on gastric emptying does not directly impact on the anastomotic stricture rate.

The size of anastomosis and its impact on anastomotic strictures is poorly understood. A meta-analysis by Honda and colleagues in 2013 including 12 randomized controlled trials demonstrated that there was no relationship between circular stapled anastomosis size and anastomotic stricture rate [9]. These results were corroborated by an elegant study by Tagkalos et al. who showed no difference between 25 and 28 mm circular stapled anastomoses [21]. In contrast, a large study from England showed a very definitive relationship of decreasing size of circular stapled anastomosis to increasing stricture rate [22]. In our cohort the only risk factor that was predictive of the development of an anastomotic stricture was stapler size. Thirty-three percent of patients who had an anastomosis size of 25mm developed an anastomotic stricture. It is likely that a slight stricture at a smaller anastomosis will have a greater impact on dysphagia-related symptoms. Having demonstrated that the stapler size has an impact on stricture formation, the surgeon should make their utmost effort to fit in the largest stapler in the proximal esophagus. We routinely dilate our esophageal stump with a 28Fr Foley catheter balloon to facilitate this.

Historical literature and our current data has shown that despite the progress made with reduction in complications and improvement in survival following esophagectomy the procedure remains plagued with a relatively high anastomotic stricture rate. It is likely that the etiology of strictures is multifactorial and they will remain difficult to prevent. Hence the focus should be on prompt and adequate treatment of strictures following esophagectomy.

Our study is a large single center study with patients operated on by only two surgeons which lends to uniformity in technique and with a standardized post-operative protocol used. All patients were extensively followed up with a proactive approach to identification and treatment of anastomotic strictures at the earliest possible stage. However we acknowledge several limitations of the study including the retrospective data analysis which may lead to selection bias due to confounding factors which are unobserved. Additionally, as the current study is based on a circular stapled anastomosis technique performed during an open operation there may be concern about the relevance of the results particularly with regard to alternative anastomotic techniques and in the context of minimally invasive or robotic surgery. However, our results remain important as majority of oesophageal resections are still performed with open technique in most centers [23, 24].

In our unit we have a driven approach to identifying post-operative dysphagia and offering contrast studies or endoscopy at the earliest possible stage. In our study the median time from surgery to first endoscopic dilatation was 2.9 months with the earliest dilatation occurring five weeks following surgery. Anastomotic strictures can occur very early after surgery and can be safely dilated. With our proactive approach for early identification and persevered treatment of strictures we were able to resolve the strictures. Anastomotic strictures do impact on post-operative quality of life but we have demonstrated that the anastomotic strictures can be effectively treated with recurrent endoscopic balloon dilatation and importantly did not lead to excess weight loss at 1-year post-operatively.

## Conclusion

Post-esophagectomy anastomotic strictures are common and not always related to anastomotic leaks or absence of pyloroplasty. Smaller anastomosis size was strongly linked with stricture formation. A driven approach with regular endoscopic balloon dilation is safe and effective in treating these strictures with no excess weight loss at 1 year once treated.

## References

[1] Rutegård M, Lagergren P, Rouvelas I, Mason R, Lagergren J (2012) Surgical complications and long-term survival after esophagectomy for cancer in a nationwide Swedish cohort study. Eur J Surg Oncol 38(7):555–561 22483704 10.1016/j.ejso.2012.02.177

[2] Low DE, Kuppusamy MK, Alderson D, Cecconello I, Chang AC, Darling G, Davies A, D’Journo XB, Gisbertz SS, Griffin SM, Hardwick R, Hoelscher A, Hofstetter W, Jobe B, Kitagawa Y, Law S, Mariette C, Maynard N, Morse CR, Nafteux P, Pera M, Pramesh CS, Puig S, Reynolds JV, Schroeder W, Smithers M, Wijnhoven BPL (2019) Benchmarking complications associated with esophagectomy. Ann Surg 269(2):291–298 29206677 10.1097/SLA.0000000000002611

[3] Briel JW, Tamhankar AP, Hagen JA, DeMeester SR, Johansson J, Choustoulakis E, Peters JH, Bremner CG, DeMeester TR (2004) Prevalence and risk factors for ischemia, leak, and stricture of esophageal anastomosis: gastric pull-up versus colon interposition. J Am Coll Surg 198(4):536–541 15051003 10.1016/j.jamcollsurg.2003.11.026

[4] Nederlof N, Tilanus HW, Tran TC, Hop WC, Wijnhoven BP, de Jonge J (2011) End-to-end versus end-to-side esophagogastrostomy after esophageal cancer resection: a prospective randomized study. Ann Surg 254(2):226–233 21725230 10.1097/SLA.0b013e31822676a9

[5] Ahmed Z, Elliott JA, King S, Donohoe CL, Ravi N, Reynolds JV (2017) Risk factors for anastomotic stricture post-esophagectomy with a standardized sutured anastomosis. World J Surg 41(2):487–497 27778075 10.1007/s00268-016-3746-0

[6] Norton BC, Papaefthymiou A, Aslam N, Telese A, Murray C, Murino A, Johnson G, Haidry R (2024) The endoscopic management of oesophageal strictures. Best Pract Res Clin Gastroenterol. 10.1016/j.bpg.2024.101899 38749578 10.1016/j.bpg.2024.101899

[7] Järvinen T, Cools-Lartigue J, Robinson E, Räsänen J, Ilonen I (2021) Hand-sewn versus stapled anastomoses for esophagectomy: We will probably never know which is better. JTCVS Open 28(7):338–352 10.1016/j.xjon.2021.07.021 PMC9390502 36003702

[8] Kamarajah SK, Bundred JR, Singh P, Pasquali S, Griffiths EA (2020) Anastomotic techniques for oesophagectomy for malignancy: systematic review and network meta-analysis. BJS Open 4(4):563–576 32445431 10.1002/bjs5.50298 PMC7397345

[9] Honda M, Kuriyama A, Noma H, Nunobe S, Furukawa TA (2013) Hand-sewn versus mechanical esophagogastric anastomosis after esophagectomy: a systematic review and meta-analysis. Ann Surg 257(2):238–48 23001084 10.1097/SLA.0b013e31826d4723

[10] Kim RH, Takabe K (2010) Methods of esophagogastric anastomoses following esophagectomy for cancer: a systematic review. J Surg Oncol 101(6):527–33 20401920 10.1002/jso.21510

[11] Liu QX, Min JX, Deng XF, Dai JG (2014) Is hand sewing comparable with stapling for anastomotic leakage after esophagectomy? A meta-analysis. World J Gastroenterol 20(45):17218–26 25493038 10.3748/wjg.v20.i45.17218 PMC4258594

[12] Tanaka K, Makino T, Yamasaki M, Nishigaki T, Miyazaki Y, Takahashi T, Kurokawa Y, Nakajima K, Takiguchi S, Mori M, Doki Y (2018) An analysis of the risk factors of anastomotic stricture after esophagectomy. Surg Today 48(4):449–454 29170824 10.1007/s00595-017-1608-5

[13] Nishikawa K, Fujita T, Yuda M, Tanaka Y, Matsumoto A, Tanishima Y, Yanaga K (2020) Early prediction of complex benign anastomotic stricture after esophagectomy using early postoperative endoscopic findings. Surg Endosc 34(8):3460–3469 31571033 10.1007/s00464-019-07123-z

[14] Wang X, Pei X, Li X, Gao M, Cheng H, Zhong H, Cao Q (2019) Predictive value of anastomotic blood supply for anastomotic stricture after esophagectomy in esophageal cancer. Dig Dis Sci 64(11):3307–3313 30632053 10.1007/s10620-018-5451-3

[15] Jogiat UM, Sun WYL, Dang JT, Mocanu V, Kung JY, Karmali S, Turner SR, Switzer NJ (2022) Gastric ischemic conditioning prior to esophagectomy reduces anastomotic leaks and strictures: a systematic review and meta-analysis. Surg Endosc 36(7):5398–5407 34782962 10.1007/s00464-021-08866-4

[16] Koshy RM, Brown JM, Chmelo J, Watkinson T, Phillips AW (2022) Anastomotic stricture after Ivor Lewis esophagectomy: An evaluation of incidence, risk factors, and treatment. Surgery 171(2):393–398 34482991 10.1016/j.surg.2021.07.034

[17] Rice TW (2006) Anastomotic stricture complicating esophagectomy. Thorac Surg Clin 16(1):63–73 16696284 10.1016/j.thorsurg.2006.02.002

[18] Blackmon SH, Correa AM, Wynn B, Hofstetter WL, Martin LW, Mehran RJ, Rice DC, Swisher SG, Walsh GL, Roth JA, Vaporciyan AA (2007) Propensity-matched analysis of three techniques for intrathoracic esophagogastric anastomosis. Ann Thorac Surg 83(5):1805–13 17462404 10.1016/j.athoracsur.2007.01.046

[19] Helminen O, Kytö V, Kauppila JH, Gunn J, Lagergren J, Sihvo E (2019) Population-based study of anastomotic stricture rates after minimally invasive and open oesophagectomy for cancer. BJS Open 3(5):634–640 31592081 10.1002/bjs5.50176 PMC6773660

[20] Na B, Kang CH, Na KJ, Park S, Park IK, Kim YT (2023) Risk factors of anastomosis stricture after esophagectomy and the impact of anastomosis technique. Ann Thorac Surg 115(5):1257–1264 36739069 10.1016/j.athoracsur.2023.01.026

[21] Tagkalos E, van der Sluis PC, Uzun E, Berlth F, Staubitz J, Gockel I, van Hillegersberg R, Lang H, Grimminger pp. (2021) The circular stapled esophagogastric anastomosis in esophagectomy: no differences in anastomotic insufficiency and stricture rates between the 25 mm and 28 mm circular stapler. J Gastrointest Surg 25(9):2242–2249 33506342 10.1007/s11605-020-04895-x PMC8484169

[22] Dresner SM, Lamb PJ, Wayman J, Hayes N, Griffin SM (2000) Benign anastomotic stricture following transthoracic subtotal oesophagectomy and stapled oesophago-gastrostomy: risk factors and management. Br J Surg 87(3):362–73 10718959 10.1046/j.1365-2168.2000.01383-5.x

[23] Park MH, Wahedally MAH, Maynard N, Crosby T, Thomas B, Trudgill N, Geisler J, Napper R. Cromwell D. National Oesophago-Gastric Cancer Audit (2022). Annual Report. London: The Royal College of Surgeons of England, 12 January 2023

[24] Griffiths EA, Collaborative OGAAO, Halle-Smith JM, Kamarajah SK, Evans RPT, Nepogodiev D, Hodson J, Bundred JR, Gockel I, Gossage JA, Isik A (2024) Predictors of anastomotic leak and conduit necrosis after oesophagectomy: Results from the oesophago-gastric anastomosis audit (OGAA). Eur J Surg Oncol 50(6):107983 38613995 10.1016/j.ejso.2024.107983

